# Coal Mine Air Pollution and Number of Children Hospitalizations because of Respiratory Tract Infection: A Time Series Analysis

**DOI:** 10.1155/2015/649706

**Published:** 2015-07-12

**Authors:** Yonglin Liu, Juan Liu, Fenglian Chen, Shamsi Bilal Haider, Qiang Wang, Fuyong Jiao, Yanmei Qiao, Yanhua Shi

**Affiliations:** ^1^Pediatrics Department, Shenmu Hospital, Shenmu, Yulin, Shaanxi 719300, China; ^2^Shenmu Hospital, Shenmu County, Yulin, Shaanxi 719300, China; ^3^Shaanxi Provincial People's Hospital, Xi'an, Shaanxi 710061, China

## Abstract

To analyze the relationship between levels of air pollution and number of children hospitalizations because of respiratory tract infection in Shenmu County, the data regarding meteorological factors, environmental pollutants, that is SO_2_ and NO_2_, Particulate Matter 10 (PM10), and hospitalizations of children less than 16 years of age was collected during the time duration of November 2009 to October 2012. Using SAS 9.3, descriptive data analysis for meteorological and environmental factors and hospital admissions were performed along with main air pollutants determination. Using the statistical software R 3.0.1, a generalized additive Poisson regression model was established, the linear fitting models of the air pollutant concentrations and meteorological factors were introduced considering the lag effect, and the relative risk of the main atmospheric pollutants on children hospitalization was evaluated. The results showed that the primary air pollutant in Shenmu County is PM10 and its Pearson correlation coefficient with Air Pollution Index (API) is 0.917. After control of long term climate trend, “week day effect,” meteorological factors, and impact of other contaminants, it was found that, on the same day and during the lag of 1 to 10 days, PM10 concentrations had no significant effect on children hospitalization rate.

## 1. Introduction

Air pollution and respiratory diseases are closely related [1] leading to the increase in hospital admissions and health care burden [2, 3]. There are severe health effects caused by burning coal [4]. According to the reports issued by the World Health Organization and by environmental groups, coal particulates pollution is estimated to shorten approximately 1,000,000 lives annually worldwide [5]. Respiratory tract infections in children have a higher prevalence [6], particularly upper respiratory tract infections, pneumonia, and bronchitis [7, 8].

Extensive research regarding adverse effects of atmospheric pollution has been done in many major cities abroad but in China such research is limited to a few big cities [9]. Shenmu County in northern Shaanxi Province has Shenfu Dongsheng coalfield, which is one of the world's largest coalfield [10], coal mining has produced a serious bituminous type atmospheric pollution [11, 12]. Therefore, it is necessary to quantitatively evaluate the Shenmu air pollution concentration and its impact on childhood hospitalization due to respiratory diseases, in order to provide the strong basis for the protection of children respiratory health. In this study, we applied the Generalized Additive Model (GAM) extended Poisson regression model to quantitatively evaluate the effects of ambient air pollutants, NO_2_, PM10, and SO_2_, on the prevalence of respiratory diseases by analyzing the time-series data of air pollutants, meteorological factors, and number of hospitalizations because of respiratory diseases in Shenmu County, Yulin City of China.

## 2. Methodology

### 2.1. Ethics Statement

This analysis is done with the approval of Shenmu County Government and Shenmu Hospital Ethics Committee and is registered at Hospital Medical Affairs Department. The Study meets all the ethical principles for medical research involving human subjects and does not invade children privacy. Verbal informed consent was taken from the Parents/guardians of all the participants who also are provided with free access to the study results. The reasons for verbal informed consent were in accordance to the regulations of Office for Human Research Protections (OHRP) [13] and are as follows:

1. Data required for this study did not involve individual identifiers.
2. The county level research participants are semiliterate and are considered unable to read the information sheet.
3. The use of official forms such as information sheets and consent forms is associated with officialdom and might be perceived by participants to be threatening and might increase their worry about the child's disease condition and treatment outcome.

A script for verbal consent including the same elements of consent as would be in a consent form was reviewed and approved by Hospital Ethics Committee and according to the Economic and Social Research Council [14] (ESRC) Framework for Research Ethics (2010) [15] the witnesses were appointed by Hospital Ethics Committee to verify and document the consent process. The Hospital Ethics Committee finally approved this consent procedure.

A joint committee comprised of Shenmu County government, Shaanxi Province Science and Technology Agency, Shenmu Hospital Ethics Committee, and Shenmu Technology Bureau further reviewed and approved all the financial and ethical guidelines.

### 2.2. Patients

Data regarding Hospitalization record of children was collected from Shenmu County Hospital and Shenmu Second Hospital during 1st November, 2009, to 30th October, 2012. The cases were selected having primary discharge diagnosis of respiratory tract infections or secondary diagnosis including the following diseases:

1. Upper respiratory tract infections including rhinitis, rhinosinusitis, nasopharyngitis, pharyngitis, epiglottitis, laryngitis, laryngotracheitis, tracheitis, otitis media, and the common cold [16, 17].
2. Lower respiratory tract infections including pneumonia and bronchitis [18].

### 2.3. Subject Factors Control

Children aging less than 16 years were selected with no other associated co morbidity, that is, obesity, physical disability, and allergic conditions including asthma. No history of passive smoking was conformed as a part of selection criterion. The children from stable socioeconomic family background were selected for the study.

### 2.4. Monitoring of Atmospheric Meteorological Factors and Major Pollutants

During the corresponding time period, daily monitoring data of atmospheric meteorological factors including average daily air temperature and relative humidity was collected from Shenmu Meteorological Bureau. Data regarding daily average atmospheric concentration of major pollutants including sulfur dioxide (SO_2_), nitrogen dioxide (NO_2_), and particulate matter (PM10) was collected from Shenmu Environmental Protection Agency. Shenmu County has total area of 7600 km^2^, population of 400000 people, and 4 air monitoring sites. Methodology described by American Heart Association was used for data collection and computation according to which exposure measurements during the study period were taken from all of the monitoring sites for Shenmu County, which provided hourly measurements data of suspended particles and gaseous pollutants. The hourly mean of each pollutant from all of the stations was calculated and then their 24-hour averages were computed [19]. Air pollutant concentration obtained from an air monitor over a specified averaging period represents the dose of the air pollutant. Air pollution index values are typically grouped into ranges. Each range is assigned a descriptor, a color code, and a standardized public health advisory.

### 2.5. Statistical Methods

The daily continuous data of air pollution and children hospitalization due to respiratory tract infections was analyzed as time series data of generalized additive model (GAM) [20]. During the corresponding study period, the SAS 9.3 statistical software was used for the descriptive analysis to understand the general characteristics and relationship of the average daily temperature, relative humidity, atmospheric SO_2_, NO_2_ and PM10 concentration along with the incidence of disease. According to the formula, the air pollution index (API) and the quantity of the primary pollutants were calculated. The main pollutants were determined through exploring and understanding the possible colinearity and simple Pearson correlation of all the variables [9, 21].

Since the number of daily respiratory diseases is countable data but relative to the whole population, it is a small probability event so the daily respiratory infections data was considered to be consistent with a Poisson distribution [22]. Based on the descriptive exploration of the data, GAM software of R 3.0.1 statistical software package was used to create generalized additive model for the Poisson data [23]. Atmospheric pollutant concentrations and meteorological factors were fitted into linear models, and considering the lag effects it was determined that the main pollutants and respiratory diseases had significant correlation and relevance. In this study, Akaike information criterion (AIC) was used for model comparison and screening [24–27].

Regression analysis was performed using the Lag effect in the health indicators of the same day in relation to the level of atmospheric pollution several days before, predicting the effect of atmospheric pollution on future health parameters. In this study, the concentration of pollutants on 1 day was set as standard, and the average of pollutant concentration of that day and the day before was set as the 1st lag. Poisson regression was performed using 1 to 10 days lag to analyze atmospheric pollutants concentration and daily hospital admissions due to respiratory causes and the relative risk of atmospheric pollutants on the incidence of children hospitalization due to respiratory infections was evaluated.

## 3. Results

### 3.1. Basic Conditions

A total of 15,323 patients got admission in both Shenmu county Hospital and Shenmu Second Hospital from 1st November 2009 to 31st October 2012. Amongst the hospitalized children, 3178 patients were of upper respiratory tract infection and 3447 patients of lower respiratory tract infection. Number of respiratory infection hospital admissions, and the daily average concentrations of air temperature, relative humidity, SO_2_, NO_2_, and PM10 are given in Table 1.

In these 3 years during a total of 1096 days, the primary pollutant during 103 days was SO_2_ accounting for 9.4%: 15 days NO_2_ accounting for 1.4% and 978 days PM10 accounting for 89.2%. So it is obvious that county air pollution was mainly PM10 based.

### 3.2. Correlation Analysis between Each of the Air Pollutants and Meteorological Factors

By the Pearson correlation coefficient table (Table 2), it can be seen that the correlation coefficient of PM10 and API is 0.917, which is significant correlation. The homology between temperature, humidity, and the three kinds of atmospheric pollutants has statistical importance but the correlation coefficient is not significant.

### 3.3. Variation Tendency between PM10, Temperature, and Respiratory Disease Incidence

**Figure 1:**
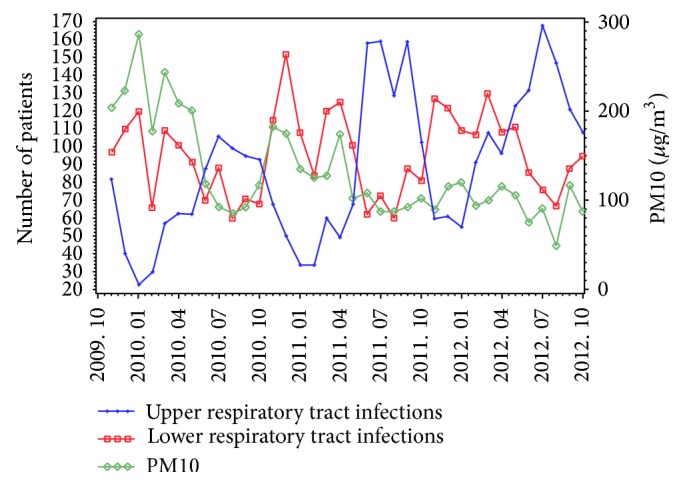
PM10 and respiratory tract infections variation trends.

**Figure 2:**
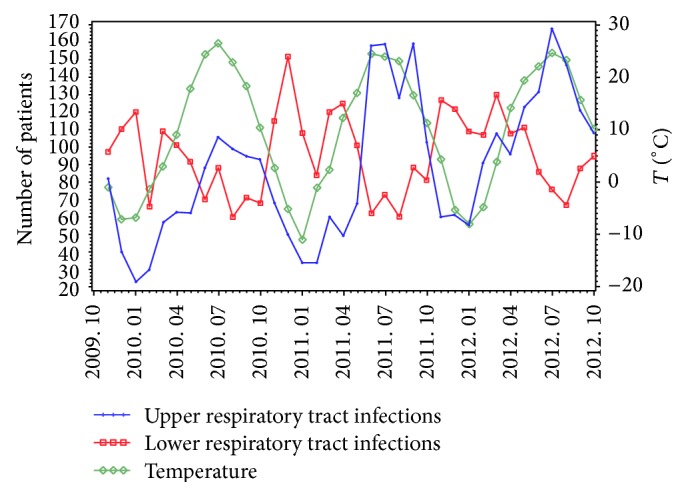
Temperature and respiratory tract infections variation trends.

PM10 is the county's major air pollutant and a statistically significant correlation exists between temperature and atmospheric pollutants. Therefore, in order to calculate the mean values for PM10, temperature, and the sum of respiratory disease cases, scatter plot was used to observe the variation tendency of these parameters. From Figure 1, it can be derived that the variation tendency of PM10 and lower respiratory tract infections is parallel but opposite to the upper respiratory tract infections. It is already known by the results of aforementioned Pearson correlation that the temperature was negatively correlated with PM10. So connecting with Figure 2 it can be easily derived that the variation tendency of temperature is opposite to lower respiratory tract infections but parallel to the upper respiratory tract infections. Thus, with the decrease in temperature, the PM10 value increases with the increase in the incidence of LRTIs. It can be seen by consolidating Figures 1 and 2 that the number of upper and lower respiratory tract infection cases show a potential shift but collectively remain unchanged.

### 3.4. Analysis of the Generalized Additive Model

On the basis of long term tendency control of the “weekday effect,” temperature, and humidity, and simultaneously considering the average lag effect, the primary pollutant PM10 was put into the model.  Table 3 is a single pollutant model having the relative risk (RR) and 95% confidence intervals (95% CI) of the daily children hospitalizations because of respiratory tract infections relative to every 10 *μ*g/m^3^ rise in the primary air pollutant, PM10 concentration. It can be seen from Table 3 that on one standard day and during the 10 days lag, PM10 concentration changes did not produce any significant change in the upper and lower respiratory tract infection hospitalization rates.

## 4. Discussion

Generalized additive model (GAM) controls the long-term variation tendency of time [28], weekday effects [29], and meteorological factors [30] and blends properties of generalized linear models with additive models [31]. GAM fits nonparametric or semi parametric additive models, supports the use of multidimensional data, supports multiple SCORE statements, and enables specification of the model degrees of freedom or smoothing parameter [32].

The study of effects of atmospheric pollution on the residents' respiratory infections has become a research focus in recent years [1, 8, 33]. GAM model has been reported to study the effects of environmental pollution on health [22, 34, 35].

Atkinson et al. found that with each 10 *μ*g/m^3^ rise of PM10, average daily number of hospitalizations of respiratory disease patients increased by 0.9% [36]. The study conducted in Hong Kong also reported that with increase in PM10 concentration, the incidence of respiratory diseases also increases, especially in children, and the average daily number of respiratory disease hospitalizations increases significantly [37]. Domestic researches conducted in Jinan city study found that average atmospheric concentrations of PM10, SO_2_, and NO_2_ and number of outpatient department respiratory diseases have obvious direct relationship [38], in which the best of PM10 lag time is 0 days. Simultaneously it was observed that combined effects of SO_2_ and PM10 caused more harm to health [39, 40].

Although this study found that the amount of atmospheric PM10 was positively related to the variation tendency of lower respiratory tract infection cases while it was conversely related to the variation tendency of upper respiratory tract infection cases, but fitting GAM model, we did not observe any significant correlation between amount of atmospheric PM10 and number of respiratory infection cases among children. Taking into account the mutual influence that may exist between pollutants; we observed both ways, that is, put PM10 alone and in combination with SO_2_ and NO_2_, into the model, but yet did not observe any significant correlation between PM10 and number of respiratory tract infection hospitalizations in children. Possible reasons for this may be as follows:

1. Hospitalized cases do not represent the total population incidence. Although air pollution affects the incidence of respiratory disease, but this effect is reflected more in outpatients as compared to the hospitalized patients because most of the patients get hospitalized when their disease becomes severe with more complications.
2. PM10 effect on respiratory infection is not strong. The study results of Beijing's Chaoyang District also suggest that PM10 concentration has no obvious impact on the health of residents [41].
3. We may consider that differences in weather characteristics between different research sites could influence the composition of the pollution mix and therefore produce different effects on human health [42].
4. The possibility of various mechanistic pathways with multifaceted interdependencies must be taken into consideration when interpreting the results. Shenmu County of Yulin city has an elevation above sea level of 1044 meters or 3425 feet [43]. So, altitude related physiologic polycythemia which is a normal adaptation to living at high altitudes [44] can also be taken into consideration while interpreting the above results, which can be a factor giving Shenmu County children more resistance against respiratory diseases caused by air pollutants. More research studies can be performed focusing on the relationship of respiratory diseases and air pollutants in different cities with different altitudes.

This study has several limitations. The first is the small number of patients available. The second limitation is the length of the data collection period, that is, only 3 years. Thirdly the organisms involved in the upper and lower RTIs corresponding to the changes in meteorological factors and pollutants are not included in the study. The results of the study provide intriguing yet compelling further research into pathophysiology that links exposure to fine particulate air pollution and respiratory diseases.

## 5. Conclusion

It is the first county level environmental research done in China and it will not only contribute to the international research data but also pave a path for further research regarding atmospheric pollution, taking into consideration different weather characteristics, composition of the pollution mix, and the physiologic differences of the residents of different research sites. In this study, based on the full access to Shenmu County's meteorological environmental information and hospitalization data of children with respiratory illness, and using the GAM model analysis, we found that Shenmu county PM10 has no significant effect on the prevalence of respiratory disease. Future studies should attempt to verify these findings and investigate in more depth the differences in magnitude of effects between different populations.

## Figures and Tables

**Table 1:** Number of respiratory infection hospital admissions, meteorological factors, and pollutant concentrations.

Variables	±s	Median	P_25_	P_75_	Maximum
Upper respiratory tract infections (10^3^)	2.9 ± 2.2	2.5	1.0	4.0	14.0
Lower respiratory tract infections (10^3^)	3.1 ± 2.0	3.0	2.0	4.0	12.0
Temperature (°C)	9.8 ± 12.0	11.4	−0.5	21.0	47.3
Relative humidity (%)	50.2 ± 17.1	49.9	38.1	62.1	83.5
SO_2_ (*μ*g/m^3^)	49.3 ± 71.0	27.0	13.0	58.0	722.0
NO_2_ (*μ*g/m^3^)	41.4 ± 23.0	38.0	27.0	51.0	149.0
PM10 (*μ*g/m^3^)	131.0 ± 93.4	110.0	78.0	154.0	971.0
API	112.1 ± 65.3	107.0	82.8	128.5	862.0

**Table 2:** Pearson correlation coefficient between air pollutants and meteorological factors.

	Temperature	Relative humidity	SO_2_	NO_2_	PM10	API
Temperature	1.000	0.098^*∗∗*^	−0.121^*∗∗*^	−0.151^*∗∗*^	−0.252^*∗∗*^	−0.185^*∗∗*^
Relative humidity		1.000	−0.042^*∗∗*^	−0.052^*∗∗*^	−0.175^*∗∗*^	−0.138^*∗∗*^

SO_2_			1.000	0.118^*∗∗*^	0.143^*∗∗*^	0.209^*∗∗*^

NO_2_				1.000	0.311^*∗∗*^	0.219^*∗∗*^

PM10					1.000	0.917^*∗∗*^

API						1.000

^*∗*^ *P* < 0.05; ^*∗∗*^ *P* < 0.01.

**Table 3:** The relative risk of hospital admissions because of respiratory tract infection with every *μ*g/m^3^ rise in PM10 concentration.

	Lag days	RR (95% CI)	*t*	*P*
Upper respiratory tract infections	0	0.997 (0.991–1.004)	−0.819	0.413
	1	0.997 (0.991–1.003)	−1.127	0.260
	2	1.000 (0.994–1.005)	−0.077	0.938
	3	0.993 (0.987–0.999)	−2.315	0.021
	4	0.996 (0.990–1.002)	−1.422	0.155
	5	0.997 (0.991–1.002)	−1.155	0.248
	6	0.999 (0.994–1.005)	−0.195	0.846
	7	1.000 (0.994–1.005)	−0.082	0.935
	8	1.003 (0.997–1.008)	0.925	0.355
	9	0.997 (0.991–1.003)	−0.905	0.366
	10	1.002 (0.996–1.007)	0.589	0.556

Lower respiratory tract infections	0	0.997 (0.992–1.002)	−1.104	0.270
	1	1.001 (0.997–1.006)	0.480	0.631
	2	1.002 (0.997–1.006)	0.760	0.448
	3	1.001 (0.996–1.005)	0.280	0.780
	4	1.001 (0.997–1.006)	0.651	0.515
	5	1.002 (0.997–1.006)	0.757	0.449
	6	0.999 (0.994–1.004)	−0.437	0.662
	7	1.003 (0.998–1.007)	1.158	0.247
	8	1.000 (0.995–1.005)	−0.011	0.991
	9	1.000 (0.995–1.004)	−0.092	0.927
	10	1.000 (0.995–1.005)	−0.007	0.995
